# Acid Leaching Extraction Mechanism of Aluminum and Iron Ions from Coal Gangue Based on CaF_2_ Assistance and Process Optimization

**DOI:** 10.3390/ma16020499

**Published:** 2023-01-04

**Authors:** Deshun Kong, Zihan Zhou, Shuojiang Song, Rongli Jiang

**Affiliations:** 1 Guizhou Provincial Key Laboratory of Coal Clean Utilization, School of Chemistry and Materials Engineering, Liupanshui Normal University, Liupanshui 553004, China; 2 School of Chemical Engineering and Technology, China University of Mining and Technology, Xuzhou 221016, China

**Keywords:** coal gangue, acid leaching, CaF_2_, extraction of aluminum and iron ions

## Abstract

To reveal how CaF_2_ improves the dissolution ratios of aluminum and iron ions in coal gangue, CaF_2_ and hydrochloric acid are used to extract Al^3+^ and Fe^3+^ from the coal gangue calcined powder. The leaching ratios of Al^3+^ and Fe^3+^ are measured, and the filter residues are analyzed by BET, XRD, and SEM. The results show that adding 3% CaF_2_ could increase the extraction ratio of Al^3+^ from 62.96% to 92.10% under optimized conditions, and that of Fe^3+^ is increased from 85.12% to 95.73%. The mechanism of CaF_2_ as an auxiliary to improve the leaching ratio of Fe^3+^ is that HF reacts with the thin layers of gangue calcined powder containing silica to form soluble SiF_4_, thus forming pores that promote the diffusion of H^+^ and inner ions, improving the leaching ratios of Al^3+^ and Fe^3+^. Finally, the CaF_2-_assisted acid leaching process is optimized. The results showed that it is efficient and feasible to extract Al^3+^ and Fe^3+^ with the assistance of CaF_2_ and that HF has a catalytic effect in the reaction system. This work provides a reference for the next step of actual production.

## 1. Introduction

Coal gangue is solid waste produced by coal mining and washing [1]. It currently accounts for the largest amount of industrial waste in China [2], and its piling pollutes the environment and produces additional negative effects [3,4,5,6]. It mainly consists of kaolinite, quartz, other clay minerals, and a minor amount of coal [7]. It is a resource that can be used, but the traditional ways of utilization are relatively simple, and the added value is not as high as when it is used to make brick, to pave roads, or make cement [8]. Coal gangue mainly contains aluminum, silicon, iron, and other components [9,10,11], which can be used to prepare new products after being extracted by certain methods [12]. Most of the gangue contains about 40–60% silica, more than 15–35% alumina, and 1–15% iron oxide [13,14]. The extraction of alumina is one of the key aims. The acid-leached filter residue can be used to prepare molecular sieves, realizing the resource utilization of coal gangue [15]. Previous studies generally used acid or alkali to extract the silica and aluminum ions [16,17,18]. However, the extraction ratio of aluminum ions was still not high, even using the secondary acid leaching method or extending time [19,20,21,22]. Adding fluorite and limestone in the calcination process to improve the activity of kaolinite can make the extraction ratio of the aluminum element reach more than 90% [23,24]; however, there are some shortcomings, such as high calcination temperature, elevated energy consumption, easy sintering, etc.

More than 10 million tons of coal gangue is produced annually, with a total of more than 200 million tons in Liupanshui city, Guizhou Province, China. Compared with the coal gangue in northern China, the gangue of this area is different from the gangue of other regions [25]. It is characterized by a high iron content, commonly from about 15% to 22% or more, but its Al_2_O_3_ content is only about 20%. The iron content is high, so the calcined powder is dark red, which makes it difficult to use in fields with elevated requirements for product whiteness. However, while extracting the iron and aluminum ions simultaneously for further use is undoubtedly an excellent idea, the different chemical activities of aluminum- and iron-containing substances in gangue, resulting in the difficulty of efficient dissolution of aluminum and iron ions, especially the low extraction ratio of aluminum ions, has been the difficulty of gangue extraction in the region. Due to the incomplete dissolution of aluminum ions, when the alkali solution is used to extract silicon ions, it leads to the formation of insoluble silicon-aluminate compounds from the residual aluminum and silicon ions [26], resulting in the reduction of the dissolution ratio of silicon ions and the waste of aluminum ions. On the other hand, the high content of aluminum and iron in the filter residue also leads to silicon-containing substances of low purity and the difficulty of their subsequent utilization [27]. Therefore, efficiently extracting aluminum and iron ions from the gangue is essential. Aguiar, E. et al. found that HF can extract the leaching ratio of metal ions, such as titanium, due to its ability to dissolve silica [28]. However, their study did not discuss the reaction between fluoride and silicon ions, nor did it discuss the influence of holes formed by silicon ions on the leaching rate of metal ions.

Compared with other F sources, CaF_2_ is cheap and easy to use. In this work, we added CaF_2_ to the acid leaching system to improve the leaching ratio of aluminum and iron ions. We discuss the mechanism by which CaF_2_ affects the leaching ratio of aluminum and iron, and finally optimizes the acid leaching conditions.

## 2. Materials and Methods

### 2.1. Materials

The coal gangue came from a coal mine in Liupanshui city. The CaF_2_ analytical purity reagent came from Tianjin (Zhiyuan Chemical Co., Ltd., Tianjin, China). The hydrochloric acid analytical purity reagent came from Chongqing (Chuandong Chemical Co., Ltd., Chongqing, China). The water was self-made deionized water.

### 2.2. Procedure

**Figure 1:**
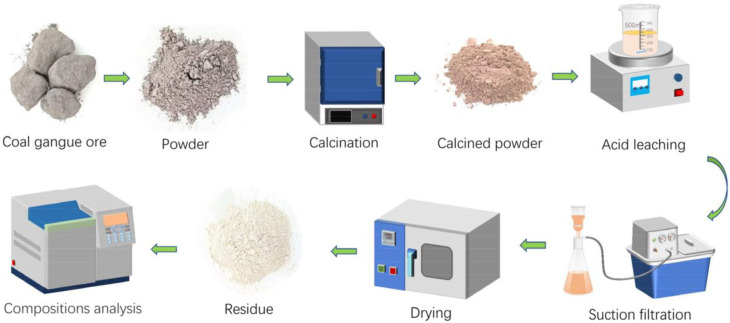
Process flow of extracting aluminum and iron ions.

As shown in Figure 1, the gangue was ground and sieved using sets of sieves of different mesh, then calcined for 1 h to obtain the calcined powder. Hydrochloric acid and CaF_2_ were added to the calcined powder by varying the values of m(CaF_2_)/m(calcined powder) (0, 0.01 g/g, 0.015 g/g, 0.02 g/g, 0.025 g/g, 0.03 g/g, 0.035 g/g, 0.04 g/g, 0.045 g/g), then reacted by water bath heating after the extraction, washing, and drying of the filter residue, and the contents of the aluminum and iron ions in the filter residue were measured. Finally, their leaching ratios were calculated.

The aluminum ion leaching ratio is calculated by the formula:

R_1_ = [(m × α_0_ − m_1_ × α_1_)/(m × α_0_)] × 100%

The iron ion leaching ratio is calculated as:

R_2_ = [(m × β_0_ − m_1_ × β_1_)/(m × β_0_)] × 100%

where R_1_: leaching ratio of aluminum element; R_2_: leaching ratio of iron element; m: mass of calcined powder; m_1_: mass of the residue, α_0_: mass fraction of aluminum element in calcined powder; α_1_: mass fraction of aluminum element in the residue; β_0_: mass fraction of iron element in calcined powder; β_1_: mass fraction of iron element in residue.

### 2.3. Instrumentation and Characterization

A 6100 type X-ray diffraction instrument (XRD, Shimadzu, Shimadzu Company, Kyoto, Japan) was used for Cu Kα (λ for Kα = 1.54059 Å), 2θ = 3–65°, with a step width of 0.02°. The main components were determined by a Supermini200 type X-ray fluorescence spectrometer (XRF, Rigaku, Rigaku Company, Tokyo, Japan). The morphologies of the materials were identified by an EVO18-type scanning electron microscope (SEM, Zeiss, Jena, Germany). The HBZS-200 type standard vibrating sieve machine was from (Hongbang Technology Company, Xingtai, China). N_2_ adsorption-desorption isotherm curves were measured on a Micromeritics TriStar II 3020 type analyzer (Mike, Mike Instruments Company, Atlantic, NJ, USA). At −196 °C, 100 mg of the sample was weighed and degassed at 150 °C for 6 h under vacuum (1 × 10^−5^ Pa) to remove adsorbed water and impurities before measurement. The specific surface area of the samples was calculated using the adsorption branch data in the relative pressure (P/P_0_) range of 0.05–0.30, based on the following Brunauer-Emmet-Teller (BET) equation: (P/P_0_)/[N(1 − P/P_0_) = [1/(N_m_C)] + [(C − 1)/N_m_C](P/P_0_)], where P_0_ is the saturation pressure; P/P_0_ is the relative pressure; C and N_m_ are constants related to the adsorption energy and monolayer loading, respectively, and N is the adsorption load. The Barrett-Joyner-Halenda (BJH) method was used to calculate the pore size distribution and obtain information on the micropore volume and total pore volume of the samples. The thermal decomposition performance of coal gangue was determined using a TGA-DSC1 type thermogravimetric analyzer (TG-DSC, Mettler Toledo, Greifensee, Switzerland). Concerning the atmosphere under the accumulation of carbon program heating oxidation, 30 °C to 1000 °C, the program heating rate was 10 °C/min.

## 3. Results and Discussion

### 3.1. Characterization of Gangue and Its Calcined Powder

**Figure 2:**
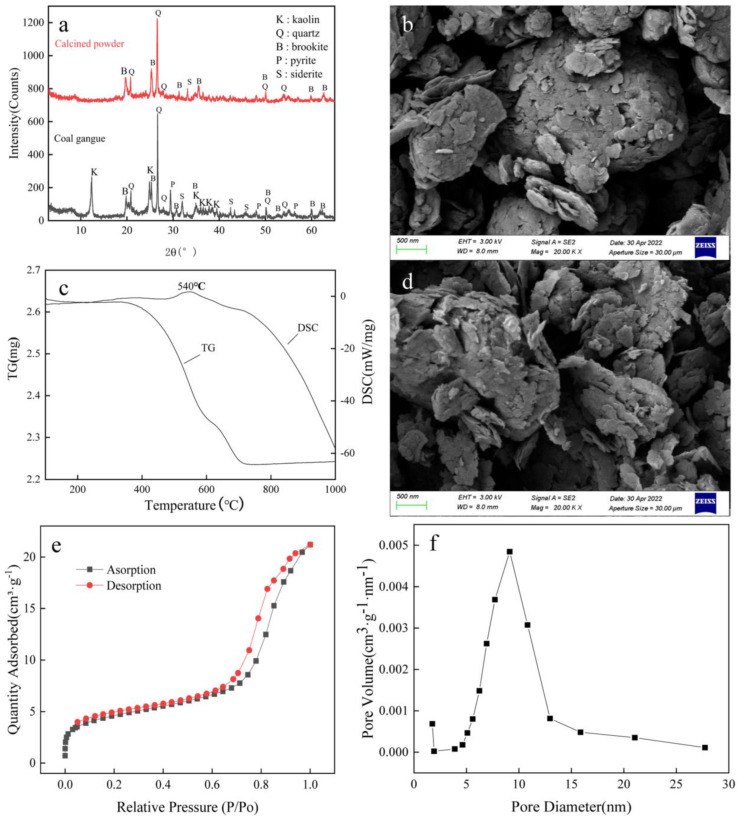
Characterization of gangue and its calcined powder. (**a**) is the XRD spectra of gangue and its calcined powder, (**b**) is the SEM diagram of raw gangue, (**c**) is the TG-DSC diagram of gangue, (**d**) is the SEM diagram of calcined powder, (**e**) is the nitrogen adsorption-desorption diagram of calcined powder, and (**f**) is the pore volume—pore size relationship of calcined powder.

Composition analysis, physical phase analysis, thermal analysis, morphological analysis, and BET analysis were performed on the gangue and its calcined powder. The composition analysis of gangue and its calcined powder were determined by XRF. The results are shown in Table 1 and Figure 2.

As can be seen from Table 1, the gangue sample had a high iron content and a low aluminum content, which belongs to the high iron and low aluminum type of gangue. The XRD spectra of the raw gangue powder and the calcined powder are shown in Figure 2a.

As can be seen in Figure 2a, the raw coal gangue powder mainly contains kaolinite and quartz, but the diffraction peak intensity of iron-bearing materials is particularly low, indicating a poor crystalline state. Under certain conditions, calcination could not only remove coal components but also improve the chemical chemistry of gangue. To determine the appropriate activation temperature, the samples were thermally analyzed. It can be seen from Figure 2c that the heat absorption valley at 100–400 °C is mainly caused by the removal of water from the gangue [29], but mass reduction is not obvious in this temperature range. When the temperature was higher than 500 °C, the kaolinite started to lose crystal water and transformed into amorphous metakaolin [30]. The chemical activity of metakaolin was higher than that of raw kaolinite, facilitating the dissolution of aluminum ions; the mass was significantly reduced, and the following reactions mainly occurred [31]:

Al_2_O_3_·2SiO_2_·2H_2_O(kaolinite) → Al_2_O_3_·2SiO_2_(metakaolin) + 2H_2_O↑ (1)

The exothermic peak near 540 °C was caused by the combustion of coal and other combustible components. To eliminate the coal component in the gangue and activate the gangue, it was decided to calcine at 700 °C for 1 h. It can be seen in Figure 2a that the main diffraction peak of kaolinite disappears after calcination, which shows that the kaolinite component in the gangue can be activated at this temperature [32]. However, the diffraction peak of quartz is still extremely strong, which is due to the inert phase of quartz, which has not undergone a phase shift. In Figure 2b, it can be seen that the original powder of gangue is agglomerated and spherical; from Figure 2d, the calcined powder undergoes interlayer peeling and is loose due to the distortion of the lamellae of silica-oxygen tetrahedra and aluminum-oxygen octahedra of kaolinite under high-temperature conditions, in which the escape of crystalline water promotes the peeling of some lamellae and makes the powder looser. The BET analysis of the calcined powder with nitrogen is shown by its hysteresis ring shape, which does not show a saturated adsorption plateau in the higher relative pressure region, which indicates that the pore structure of the calcined powder is very irregular; these pores are mainly composed of slits, cracks, and wedges of fragments, etc. Its BET-specific surface area is 15.6279 m²/g, and its maximum pore volume is at 9.12103 nm, corresponding to a pore volume of 0.00485 cm^3^g^−1^nm^−1^.

The BET analysis of calcined powder with N_2_ is shown by its hysteresis ring shape, which does not show a saturated adsorption plateau in the higher relative pressure region, indicating that the pore structure of calcined powder is very irregular. These pores are mainly composed of slits, cracks, wedge structures of fragments, etc. [33]. Its BET-specific surface area is 15.6279 m²/g, and its maximum pore capacity is at 9.12103 nm, corresponding to a pore capacity of 0.00485 cm^3^g^−1^nm^−1^.

### 3.2. Leaching of Aluminum and Iron Ions without CaF_2_

**Figure 3:**
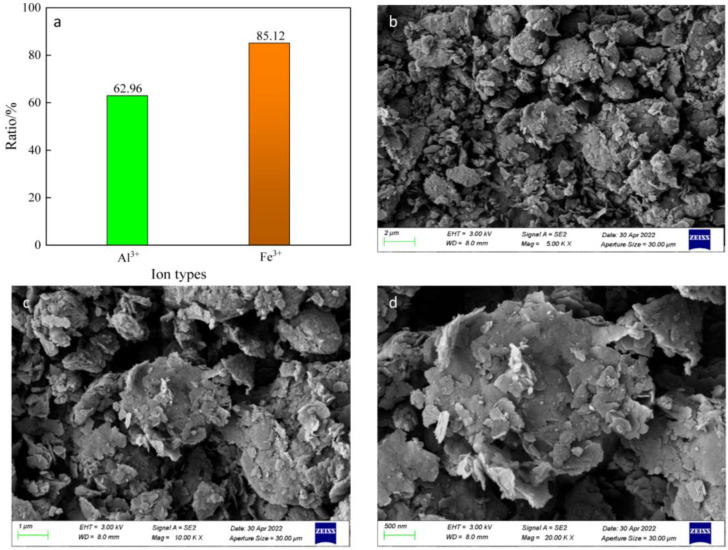
Leaching ratio and SEM image of direct acid leached residue. (**a**) is the leaching ratios of aluminum and iron ions, (**b**) is an SEM image of the acid leached residue, and (**c**,**d**) are enlarged images of the central part of Figure 2b.

Under the reaction conditions of gangue particle size <75 μm, the liquid–solid ratio of 3.5, 20% hydrochloric acid, 94 °C, 500 r/min stirring for 180 min, then the leaching ratios of aluminum and iron ions are measured, and morphological analysis are performed. The results are shown in Figure 3.

It was determined that the leaching ratio of aluminum ions was 62.96%, and the leaching ratio of iron ions was 85.12% under this condition. The chemical activity of the calcined gangue raw material was enhanced compared with that of the raw gangue [34]. As shown in Figure 3, there is partial interlayer peeling in the calcined powder particles in the form of crumbs, which favors the diffusion of ions. During acid leaching, Al_2_O_3_ and Fe_2_O_3_ in calcined powder reacted with HCl to form AlCl_3_ and FeCl_3_, and the reactions are as follows:

6HCl + Al_2_O_3_ = 2AlCl_3_ + 3H_2_O (2)

6HCl + Fe_2_O_3_ = 2FeCl_3_ + 3H_2_O (3)

2HCl + FeCO_3_ = FeCl_2_ + H_2_O + CO_2_↑ (4)

However, direct acid leaching without CaF_2_ results in low leaching ratios of aluminum and iron ions. In this study, we wanted to obtain a higher leaching ratio. For this purpose, we added CaF_2_ to the system to increase the leaching ratio of aluminum and iron ions.

### 3.3. Mechanism by Which CaF_2_ Affects Al-Fe Leaching Ratio

**Figure 4:**
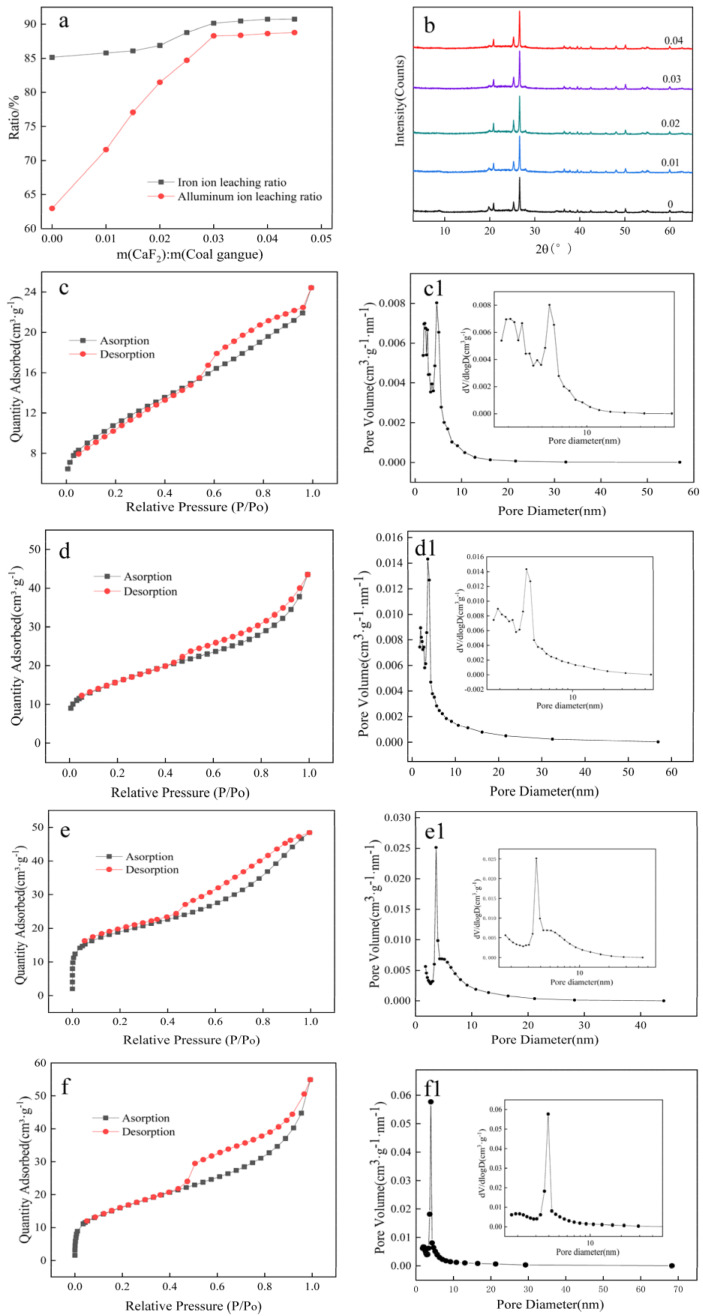
Effect of different additions of CaF_2_ on the adsorption quantity and pore volume of the residues. Adsorption: N_2_ adsorption curve of the sample; Desorption: N_2_ desorption curve of the sample. (**a**) are the relationships between CaF_2_ addition and Al-Fe leaching ratios, (**b**) are the XRD spectra of the filter residues after adding different CaF_2_, and (**c**,**c1**,**d**,**d1**,**e**,**e1**,**f**,**f1**) are the BET plots of the acid leaching filter residues after adding 0, 0.01 g/g, 0.02 g/g, and 0.03 g/g CaF_2_, respectively.

The aluminum and iron leaching ratios under different m(CaF_2_)/m(calcined powder) ratio conditions are shown in Figure 4a.

As can be seen in Figure 4a, the extraction ratio of Al and Fe ions increased with the increase of CaF_2_ dosage, and the increase of Al and Fe ions extraction ratios tended to slow down after the increase of m(CaF_2_)/m(calcined powder) to 0.03 g/g. The XRD spectra of the filter residue (Figure 4b) show that although different amounts of CaF_2_ were added, the XRD spectra of the filter residues are highly similar, with quartz as the main component. Adding CaF_2_ does not significantly change the main physical phase of the residues. To further understand how CaF_2_ affects the leaching ratio of aluminum and iron ions, the acid leaching filter residue with m(CaF_2_)/m(calcined powder) of 0, 0.01 g/g, 0.02 g/g, and 0.03 g/g was selected for the BET test, and the results correspond to Figure 4c,(c1),d,(d1),e,(e1),f,(f1), respectively.

As shown by the hysteresis ring shapes in Figure 4c–f these hysteresis rings do not exhibit saturated adsorption plateaus at higher relative pressures, indicating that the pore structure of this filter residue is irregular. These pore structures consist of biotite fragments in the residue, fissures between fragments, wedge structures, and newly formed pores. With the increase of CaF_2_ dosage, its BET-specific surface area gradually increased. The pore capacity also gradually increased, and the maximum pore capacity corresponding to the pore size increased, respectively. Their BET-specific surface area was 37.7640 m²/g, 55.1992 m²/g, 58.1395 m²/g, and 64.1203 m²/g, and its corresponding maximum pore capacity was 0.00485 cm^3^g^−1^nm^−1^, 0.00894 cm^3^g^−1^nm^−1^, 0.00666 cm^3^g^−1^nm^−1^, 0.0577 cm^3^g^−1^nm^−1^, respectively. The pore diameters where the largest pore volumes occurred were 4.64899 nm, 1.91323 nm, 1.91514 nm, and 3.93758 nm, respectively.

**Figure 5:**
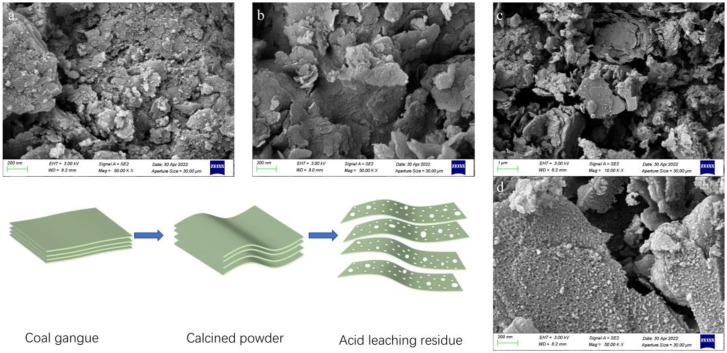
Mechanical diagram of CaF_2_ to improve the leaching ratios of aluminum and iron ions. (**a**) is an SEM image of raw coal gangue, (**b**) is an SEM image of calcined powder, (**c**) is an SEM image of acid leaching residue, and (**d**) is an enlarged view of the middle part of (**c**).

We found that the BET-specific surface area and pore volume increased after adding CaF_2_, but the pore diameter corresponding to the maximum pore volume decreased, suggesting a slight amount of fresh pore formation in the residue. To explore this issue, the acid leaching filter slag with raw coal gangue ore, calcined powder, m(CaF_2_)/m(calcined powder) of 0.03 g/g was selected. SEM analyses were performed, corresponding to Figure 5a–c. As seen in Figure 5b, after calcination, the calcined powder is flakier than the raw gangue (Figure 5a), and the acid leaching filter residue is still flaky and shows fragmentation. The central part of Figure 5c was enlarged to obtain picture Figure 5d, in which numerous small pores are visible on the lamellae. It can be seen that fresh pores were actually created after adding CaF_2_. The larger the size and capacity of the new pores, the more CaF_2_ was added, consistent with the results of the BET analysis.

In contrast, the same enlargements of Figure 5a,b do not show these little holes. These little holes are mainly caused by the removal of silicon, aluminum, and iron atoms, because the central role of the co-solvent CaF_2_ is to provide F^-^ to form H_2_SiF_6_ and SiF_4_ with SiO_2_, which is soluble in water, and the reaction equations are as follows [35]:

CaF_2_ + 2HCl = CaCl_2_ + 2HF (5)

SiO_2_ + 4HF = SiF_4_ + 2H_2_O (6)

SiF_4_ + 3H_2_O = H_2_SiO_3_ + 4HF (7)

From Equation (7), it is clear that SiF_4_ in an aqueous solution can undergo a hydrolysis reaction to produce HF, which further reacts with SiO_2_ to produce a certain catalytic effect. Through comprehensive analyses of the above, this study presents the extraction mechanism of aluminum and iron in coal gangue, as shown in Figure 5.

The gangue is mainly composed of inorganic minerals such as kaolinite. The main component, kaolinite, is a regular lamellar structure composed of a single layer of aluminum-oxygen octahedron and silicon tetrahedron and exhibits high lattice energy (45.30 kJ/mol) and a stable structure [36]. As shown in Figure 5, under calcination conditions, its lamellar crystal structure is distorted and deformed by heat to remove lattice water, resulting in amorphous metakaolin (the main component of gangue calcination powder, the silicon and aluminum layers are distorted) [37], in which silicon, aluminum, iron, and other components enter the solution under the action of hydrogen ions and fluorine ions to form pores, and the added fluorine ions dissolve. The reaction between coal gangue and hydrochloric acid is a non-catalytic reaction, which starts from the surface of the gangue and gradually penetrates the interior of the mineral [38], but the layer of silica tetrahedron hinders the penetration of hydrogen ions. When CaF_2_ is added, the HF reacts with the silicon component, which promotes the generation of pores. The large number of pores formed on the sheet layer is conducive to the entry of hydrogen ions and the outward diffusion of ions in the inner layer. These formed pores facilitate the entry of hydrogen ions and their reaction with substances containing aluminum and iron and facilitate the diffusion and dissolution of aluminum and iron ions. The more pores form in the filter residue, the more conducive to the dissolution of aluminum and iron ions. Hence, the additional CaF_2_ enhances the dissociation ratio of aluminum and iron ions.

### 3.4. Optimization of Conditions for Acid Leaching Process

**Figure 6:**
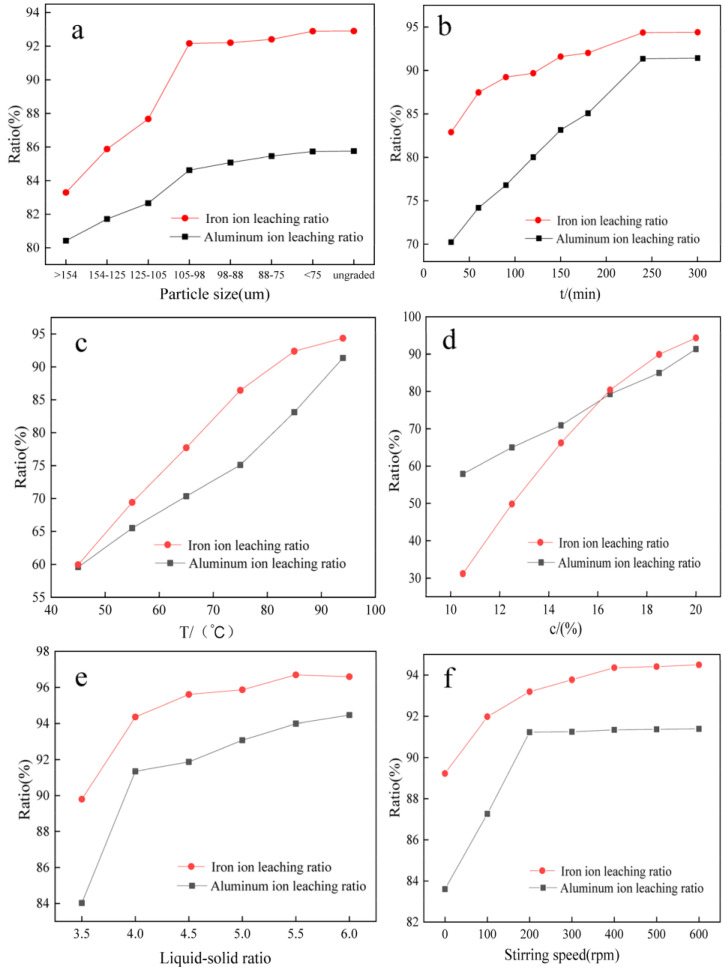
Effect of other process conditions on the leaching ratios of aluminum and iron ions. (**a**) is the relationship between particle size and leaching ratio, (**b**) is the relationship between acid leaching time and leaching ratio, (**c**) is the relationship between acid leaching temperature and leaching ratio, (**d**) is the relationship between hydrochloric acid concentration and leaching ratio, (**e**) is the relationship between liquid-solid ratio and leaching ratio, and (**f**) is the relationship between stirring speed and leaching ratio.

Other major factors that affect the leaching ratios of aluminum and iron ions include particle size, acid leaching time, acid concentration, liquid-to-solid ratio, stirring ratio, etc., which are optimized using the single-factor experiments described below. The results are shown in Figure 6.

#### 3.4.1. Effect of Gangue Particle Size on the Leaching Ratio

To each 10.00 g of different particle sizes of coal gangue, calcined powder is added m(CaF_2_)/m(calcined powder) = 0.03 g/g of CaF_2_ in the hydrochloric acid concentration of 20%, liquid–solid ratio of 4.0 mL/g, 500 r/min stirring, acid leaching temperature of 94 °C, and time of 180 min, to investigate the effect of calcined powder particle size on the leaching ratio of aluminum and iron ions. The results are shown in Figure 6a. As seen in Figure 6a, when the particle size of the calcined powder is larger, the leaching ratio is lower. The smaller the particle size, the more favorable it is to the leaching of metal ions when the particle size of 98–88 μm (after 160 mesh sieve), aluminum and iron ions leaching ratio of 85.08%, 92.02%, respectively, because the smaller the particle size, the larger the specific surface area, acid leaching particles and H^+^ can be more fully in contact, which is conducive to the chemical reaction, continue to reduce the particle size will increase energy consumption and difficult to sieve due to agglomeration of small particles. It is difficult to increase the leaching ratio, so the particle size of 98–88 μm was chosen.

#### 3.4.2. Effect of Acid Leaching Time on the Leaching Ratio

The effect of different acid leaching times (30 min, 60 min, 90 min, 120 min, 150 min, 180 min, 240 min, and 300 min) on the leaching ratios of aluminum and iron ions was investigated by selecting gangue calcined powder with a particle size of 98–88 μm and other conditions as in Section 3.4.1. The results are shown in Figure 6b. With the extension of time, Al_2_O_3_, Fe_2_O_3_, and FeCO_3_ kept reacting with hydrochloric acid, so the leaching ratios increased with the increase in reaction time. The leaching ratio of aluminum and iron reached 91.35% and 94.36% when the reaction time was 240 min, and the increase in the leaching ratio of aluminum and iron was no longer significant when the acid leaching time was extended.

#### 3.4.3. Influence of Acid Leaching Temperature on the Leaching Ratio

The reaction time was determined to be 240 min, and other conditions were the same as in Section 3.4.2. The effect of different acid leaching temperatures (45 °C, 55 °C, 65 °C, 75 °C, 85 °C, 94 °C) on the leaching ratio of aluminum and iron ions was investigated, and the results are shown in Figure 6c. At 94 °C, the aluminum leaching ratio was 89.50%, and the iron leaching ratio was 94.66%. The higher the temperature, the greater the rate of the chemical reaction. At the same temperature, the iron leaching ratio was higher than the aluminum leaching ratio, indicating that iron ions are easier to dissolve than aluminum ions in the acid leaching process.

#### 3.4.4. Effect of Hydrochloric Acid Concentration on the Leaching Ratio

The acid leaching temperature selected was 94 °C, and other conditions were the same as in Section 3.4.3. The effect of different hydrochloric acid concentrations (10.5%, 12.5%, 14.5%, 16.5%, 18.5%, 20.0%) on the leaching ratios of aluminum and iron ions were investigated, and the results are shown in Figure 6d. As seen in Figure 6d, the leaching ratios of aluminum and iron ions increase rapidly with increasing hydrochloric acid concentration, and a continued increase in concentration increases HCl volatilization. When the concentration of hydrochloric acid was 20%, the leaching ratios of aluminum and iron ions were 91.35% and 94.36%, respectively. However, higher hydrochloric acid concentrations at high temperatures tended to cause other volatilization of HCl, resulting in a wastage of hydrochloric acid, so 20% hydrochloric acid was chosen as the suitable concentration for acid leaching.

#### 3.4.5. Effect of Liquid–Solid Ratio on the Leaching Ratio of Aluminum and Iron

Other conditions were the same as in Section 3.4.4. The effect of different liquid–solid ratios (3.5 mL/g, 4 mL/g, 4.5 mL/g, 5 mL/g, 5.5 mL/g, 6 mL/g) on the leaching ratios of aluminum and iron ions were investigated, and the results are shown in Figure 6e. Figure 6e shows that the leaching ratio of aluminum and iron increases rapidly when the liquid-solid ratio is 3.5–4.0 mL/g. At a liquid–solid ratio of 4.0 mL/g, the leaching ratio of aluminum ions was 91.35%, and the leaching ratio of iron ions was 94.36%; at the liquid–solid ratio of 4.5–6.0 mL/g, the leaching ratio of aluminum and iron increased slowly. Because the liquid–solid ratio increased continuously, and the ratio of hydrogen ion concentration decreased gradually, the reaction ratio of calcined powder and hydrogen ion increased, and more aluminum and iron ions were dissolved. However, too large a liquid–solid ratio causes a great excess of acid resulting in waste, so the appropriate liquid–solid ratio was determined to be 4.0 mL/g.

#### 3.4.6. Effect of the Stirring Speed on the Leaching Ratio

Other conditions were the same as in Section 3.4.4. The effect of different stirring speeds (0, 100 r/min, 300 r/min, 400 r/min, 500 r/min, and 600 r/min) on the leaching ratios of Al^3+^ and Fe^3+^ were investigated. In Figure 6f, it can be seen that stirring accelerates the diffusion rate of ions and enhances the mass transfer process; the greater the stirring rate, the higher the leaching ratio. After reaching 500 r/min, the leaching ratios of aluminum and iron ions increased slightly, but still increased, and continuing to increase the speed results in waste, so 600 r/min is sufficient for extraction.

By exploring the acid leaching process conditions, the optimized process conditions of gangue acid leaching were obtained as follows: m(CaF_2_)/m(calcined powder) = 0.03 g/g, a gangue particle size of 98–88 μm, a reaction temperature of 94 °C, an acid leaching time of 240 min, a hydrochloric acid concentration of 20%, a liquid–solid ratio of 4.0, and stirring at 600 r/min. By the optimized process for acid leaching of coal gangue, the aluminum leaching ratio was 92.10%, and the iron leaching ratio was 95.73%.

## 4. Conclusions

1. The mechanism of CaF_2_ as an auxiliary to improve the leaching ratios of aluminum and iron ions is that HF reacts with the flake gangue calcined powder layer containing silicon to generate soluble SiF_4,_ thus forming pores. These pores are conducive to the diffusion of hydrogen ions and inner metal ions, so the dissolution ratio of aluminum and iron is improved.
2. After adding CaF_2_ to the acid leaching system, the specific surface area and pore volume of the filter residue increased with the increase of the added amount and the increase of the pore volume. HF has a catalytic effect on the reaction system.
3. In the system of extracting aluminum and iron ions by acid leaching with hydrochloric acid, adding CaF_2_ with m(CaF_2_)/m(calcined powder) = 0.03 g/g can increase the extraction ratio of aluminum ions from 62.96% to 92.10%, and iron ion from 85.12% to 95.73%. Under optimized conditions, the auxiliary of CaF_2_ is effective in improving the leaching ratios of aluminum and iron ions.

## Figures and Tables

**Table 1:** Composition analysis of gangue and its calcined powder.

	S_i_O_2_	Al_2_O_3_	Fe_2_O_3_	TiO_2_	CaO	SO_3_	K_2_O	P_2_O_5_	Loss
Gangue	44.23	18.78	12.46	3.98	2.40	0.30	1.24	0.30	16.31
Calcined powder	52.33	22.21	14.74	4.70	2.84	0.35	1.47	0.35	1.01

## Data Availability

The data presented in this study are available on request from the corresponding author.
